# Uncovering a causal connection between the *Lachnoclostridium* genus in fecal microbiota and non-alcoholic fatty liver disease: a two-sample Mendelian randomization analysis

**DOI:** 10.3389/fmicb.2023.1276790

**Published:** 2023-12-12

**Authors:** Wanhui Dai, Dandong Cai, Shuai Zhou, Ang Li, Jinsong Xie, Jie Zhang

**Affiliations:** ^1^Department of Clinical Laboratory, The Second Hospital of Nanjing, Nanjing University of Chinese Medicine, Nanjing, China; ^2^Department of Neurology, The Fifth People’s Hospital of Huai’an, Huai’an, China; ^3^Department of Endocrinology, Affiliated Huai’an Hospital of Xuzhou Medical University, Huai’an, China

**Keywords:** non-alcoholic fatty liver, gut microbiota, Mendelian randomization, causal effects, *Lachnoclostridium* genus

## Abstract

**Background:**

Previous observational studies have indicated that an imbalance in gut microbiota may contribute to non-alcoholic fatty liver disease (NAFLD). However, given the inevitable bias and unmeasured confounders in observational studies, the causal relationship between gut microbiota and NAFLD cannot be deduced. Therefore, we employed a two-sample Mendelian randomization (TSMR) study to assess the causality between gut microbiota and NAFLD.

**Methods:**

The gut microbiota-related genome-wide association study (GWAS) data of 18,340 individuals were collected from the International MiBioGen consortium. The GWAS summary data for NAFLD from the Anstee cohort (1,483 cases and 17,781 controls) and the FinnGen consortium (894 cases and 217,898 controls) were utilized in the discovery and verification phases, respectively. The inverse variance weighted (IVW) method was used as the principal method in our Mendelian randomization (MR) study, with sensitivity analyses using the MR-Egger, weighted median, simple mode, and weighted mode methods. The MR-Egger intercept test, Cochran’s *Q* test, and leave-one-out analysis were conducted to identify heterogeneity and pleiotropy. Moreover, a fixed-effect meta-analysis was conducted to verify the robustness of the results.

**Results:**

The gene prediction results showed that at the genus level, four gut microbiota were causally associated with NAFLD in the GWAS conducted by Anstee et al. The relative abundance of *Intestinimonas* (OR: 0.694, 95%CI: 0.533–0.903, *p* = 0.006, IVW), *Lachnoclostridium* (OR: 0.420, 95%CI: 0.245–0.719, *p* = 0.002, IVW), and *Senegalimassilia* (OR: 0.596, 95%CI: 0.363–0.978, *p* = 0.041, IVW) was negatively associated with NAFLD. The relative abundance of *Ruminococcus1* (OR: 1.852, 95%CI: 1.179–2.908, *p* = 0.007, IVW) was positively correlated with NAFLD. Among them, the *Lachnoclostridium* genus was validated in FinnGen GWAS (OR: 0.53, 95%CI: 0.304–0.928, *p* = 0.026, IVW). The *Lachnoclostridium* genus was also significantly associated with NAFLD risk in the meta-analyses (OR: 0.470, 95%CI: 0.319–0.692, *p* = 0.0001, IVW). No heterogeneity or pleiotropy was observed.

**Conclusion:**

This study provided new evidence of the relationship between the *Lachnoclostridium* genus and NAFLD, suggesting that augmentation of the relative abundance of the *Lachnoclostridium* genus through the oral administration of probiotics or fecal microbiota transplantation could be an effective way to reduce the risk of NAFLD.

## 1 Introduction

Non-alcoholic fatty liver disease (NAFLD) is a common chronic liver disease that affects 25% of the global population and incurs heavy economic costs on society (Younossi et al., 2016; Friedman et al., 2018). Obesity, metabolic disorders, or genetic factors contribute to the occurrence and development of NAFLD. Owing to the prevalence of obesity and diabetes, the disease burden of NAFLD is expected to increase by 2-fold to 3-fold by 2030 in Western countries and some Asian regions (Estes et al., 2018). Ishtiaq et al. demonstrated that the activation of peroxisome proliferator-activated receptor gamma (PPARγ) can exert anti-inflammatory activity by interleukin (IL)-33 expression, reducing tumor necrosis factor-alpha (TNF-α) expression, promoting storage of fatty acids as triglycerides, and inhibiting ectopic fat accumulation, which may improve NAFLD (Ishtiaq et al., 2022). Moreover, the pan-PPAR agonists have shown promising clinical outcomes in the phase 2b trial (Francque et al., 2021). Additionally, pomegranate peel extract and quercetin can treat liver injury induced by excessive oxidative stress through their antioxidant and anti-inflammatory activities (Murtaza et al., 2021). However, no approved drugs are currently available for the treatment of NAFLD. Thus, it is crucial to identify effective ways to prevent NAFLD and reduce its significant economic burden (Iruzubieta et al., 2023; Stepanova et al., 2023).

Gut microbiota plays an important role in the pathophysiology of metabolic diseases through the gut-liver axis (Aron-Wisnewsky et al., 2020). Animal studies have suggested a potential causal role of gut microbiota in NAFLD (Le Roy et al., 2013). Rashid et al. proved that probiotics might possess therapeutic potential in ameliorating high fat high sugar diet-associated alterations in metabolic profile and oxidative stress markers in rats, further suggesting the relationship between the gut microbiota and NAFLD (Rashid et al., 2020). Tiphaine et al. found that direct fecal microbiota transplantation (FMT) (from weight-matched obese mice with or without steatosis to germ-free recipients) replicated the NAFLD alterations (Le Roy et al., 2013). Additionally, in patients with NAFLD, the phylum Proteobacteria is more abundant (Grabherr et al., 2019), while at the family level, Rikenellaceae and Ruminococcaceae are decreased and Enterobacteriaceae is increased (Raman et al., 2013; Zhu et al., 2013). The gut microbiota can also alter the metabolism of lipids, glucose, and bile acids through its metabolites and induce increased intestinal permeability and inflammation, thereby affecting the development of NAFLD. In summary, a growing number of studies have shown that alterations in gut microbiota may have a causal relationship with NAFLD risk. Nevertheless, existing research has limitations, including the gap between human and animal studies and the inherent defects of observational studies, rendering the real causal nature between gut microbiota and NAFLD unclear and in need of further elucidation.

Mendelian randomization (MR) is a novel method that employs genetic variants as instrumental variables (IVs) to estimate the causal relationship between exposure and the clinical outcome of interest (Boehm and Zhou, 2022; Richmond and Davey Smith, 2022). The MR method is analogous to a randomized controlled trial (RCT), in which genetic alleles are randomly allocated at conception, and is generally not susceptible to confounding or reverse causation (Didelez and Sheehan, 2007). In this study, we first performed a two-sample MR approach to assess the causal relationship between gut microbiota and NAFLD in two independent population-scale genome-wide association studies (GWAS) data for NAFLD. The inverse variance weighted (IVW) method was used as the principal method in our MR study, with sensitivity analyses using the MR-Egger, weighted median, simple mode, and weighted mode methods. Moreover, we conducted a meta-analysis to further demonstrate the robustness of the causal relationship between the *Lachnoclostridium* genus and NAFLD.

## 2 Materials and methods

### 2.1 Study design

**Figure 1:**
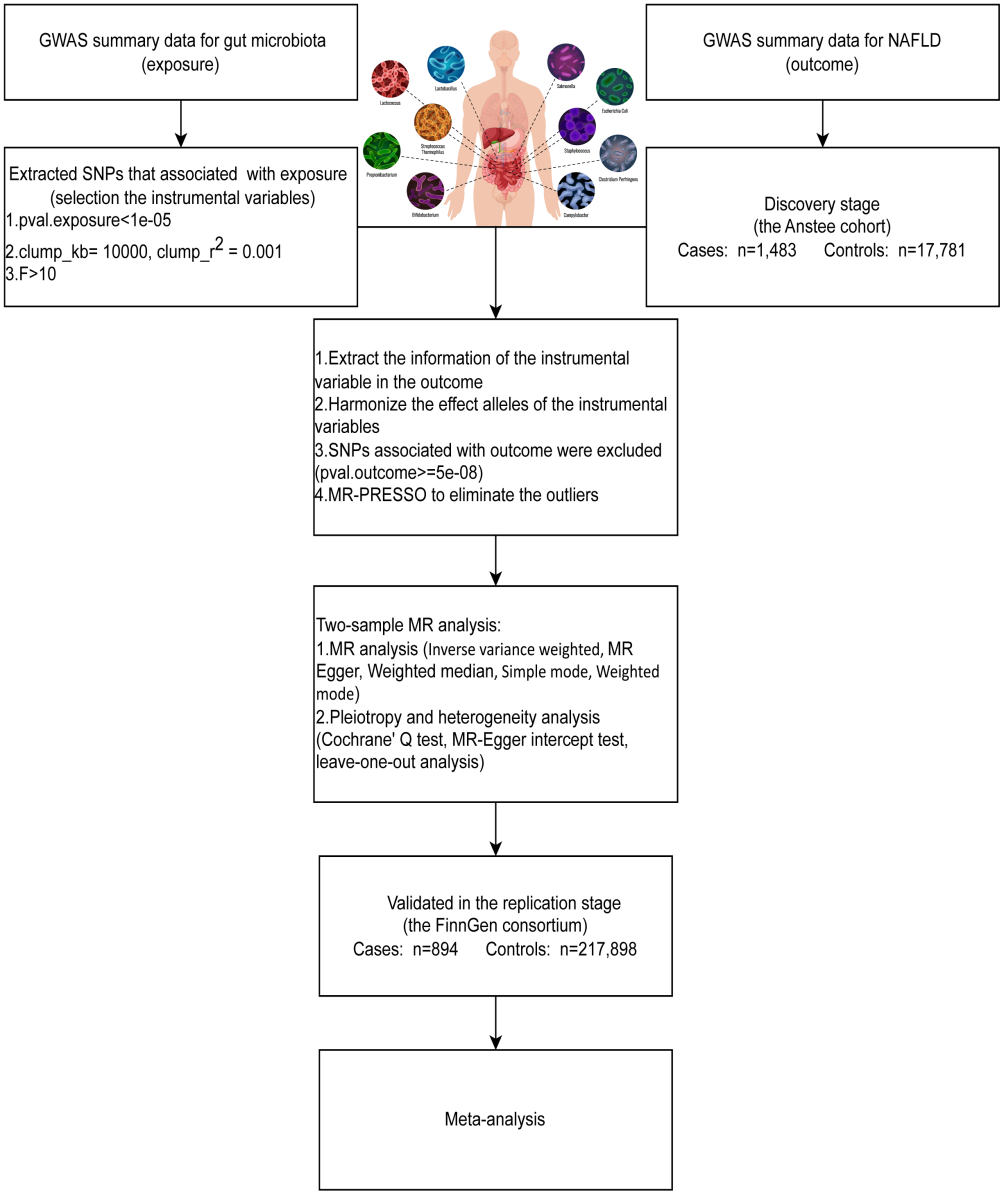
MR study design of this study.

To assess the causal relationship between gut microbiota and NAFLD, we first performed a two-sample MR (TSMR) using GWAS summary data for NAFLD from the Anstee cohort (discovery stage) and the FinnGen consortium (replication stage). To increase the power of the analysis, we combined two independent population-scale GWAS data for NAFLD to conduct a fixed-effects meta-analysis. An overview of the study design is shown in Figure 1. The causal estimates derived from MR analysis must satisfy three core assumptions: (1) relevance assumption: the genetic variants are strongly associated with the exposure; (2) independence assumption: the genetic variants are not associated with any confounders; (3) exclusion-restriction assumption: the genetic variants affect the outcome solely through the exposure.

### 2.2 Data sources for gut microbiota and NAFLD

The summary-level GWAS data of gut microbiota, obtained from the MiBioGen consortium, was used to screen the single nucleotide polymorphisms (SNPs) that were significantly associated with the gut microbiota. The MiBioGen consortium consists of 18,340 European ancestry participants from 24 cohorts with 211 taxa: 131 genera (12 unknown genera), 35 families (3 unknown families), 20 orders, 16 classes, and 9 phyla (Kurilshikov et al., 2021). Detailed information on the analyzed taxa is presented in Supplementary Table S1. The GWAS summary data for NAFLD from the Anstee cohort with 1,483 cases and 17,781 controls were used as the discovery dataset (Anstee et al., 2020). The validation dataset for NAFLD (finn-b-NAFLD), which included 894 European cases and 217,898 European controls, was obtained from the IEU OpenGWAS project.^1^

### 2.3 Instrumental variable selection

Due to the minimal number of loci found for gut microbiota, SNPs associated with gut microbiota (*p* < 1*10^−5^) were selected as instruments in our MR analysis. SNPs with *p* < 1*10^−5^ were regarded as the optimal threshold in most gut microbiota-related MR research (Sanna et al., 2019; Luo et al., 2022, 2023). Additionally, an increased number of eligible SNPs could be used for the sensitivity analysis. To identify the independent SNPs assorted randomly during gestation, we then conducted a clumping process (*r*^2^ < 0.001, region size = 10,000 kb) to assess the linkage disequilibrium (LD) by using the PLINK (version 1.9) (Purcell et al., 2007). The parameter values were set according to the previously published studies (Luo et al., 2023; Li et al., 2023a). After IVs were retrieved from the NAFLD GWAS data, we then removed the SNPs that were significantly associated with NAFLD (*p* < 5*10^−8^). After the harmonization process, F-statistics were calculated for each SNP to evaluate the strength of the IVs. The F-statistics of SNP < 10 indicated a potentially weak instrument. Weak IVs may lead to a decrease in the efficiency of statistical tests and result in bias, which needs to be eliminated (Brion et al., 2013). We also utilized the outlier test of the MR-PRESSO (version 1.0) package in R to eliminate outliers.

### 2.4 Mendelian randomization analysis

The inverse variance weighted (IVW) method was used as the main method and supplemented by four sensitivity analyses, including MR-Egger, weighted median, weighted mode, and simple mode, to evaluate the causal relationship between gut microbiota and NAFLD (Burgess et al., 2013). Cochran’s *Q* test was used to determine whether the SNPs were heterogeneous. The IVW random-effect mode was used when heterogeneity existed, as indicated by a *p*-value <0.05 in Cochran’s *Q* test. Conversely, if the *p*-value was ≥0.05 in Cochran’s *Q* test, it signified no heterogeneity (Wu et al., 2020; Li et al., 2023b). The MR-Egger intercept test was conducted to assess horizontal pleiotropy, and a *p*-value ≥0.05 indicated no evidence of horizontal pleiotropy. On the other hand, a *p*-value <0.05 suggested the presence of horizontal pleiotropy, potentially introducing bias in MR analysis (Jiang et al., 2023; Xie et al., 2023). In addition, leave-one-out analyses were applied to assess whether the variant drove the association between the exposure and the outcome variable. If the IVW method result was significant (*p* < 0.05) and the beta values obtained by the five methods were in the same direction without pleiotropy and heterogeneity, it could be considered a positive result (Chen et al., 2020; Wang et al., 2023). In addition, we performed a fixed-effects meta-analysis to further demonstrate the robustness of the causal relationship.

All statistical analyses were conducted with the “TwosampleMR” (version 1.0), “MR-PRESSO” (version 0.5.6), and “Meta” packages (version 6.5-0) in R 4.1.2. The threshold for the significance of IVW, MR-Egger, weighted median, simple mode, and weighted mode methods in the MR study was *p* < 0.05. The threshold for the significance of other analyses has been specified in the corresponding position.

## 3 Results

### 3.1 Identification and validation of the causal effect of gut microbiota on NAFLD

We first screened the IVs of 196 gut microbiota separately. Following the IV selection protocols, 2,213 SNPs for gut microbiota traits with NAFLD were finally identified in this study. The F-statistics for the IVs significantly associated with gut microbiota were all larger than 10, indicating that there was no evidence of weak instrument bias. The details of the selected IVs are presented in Supplementary Table S2.

**Figure 2:**
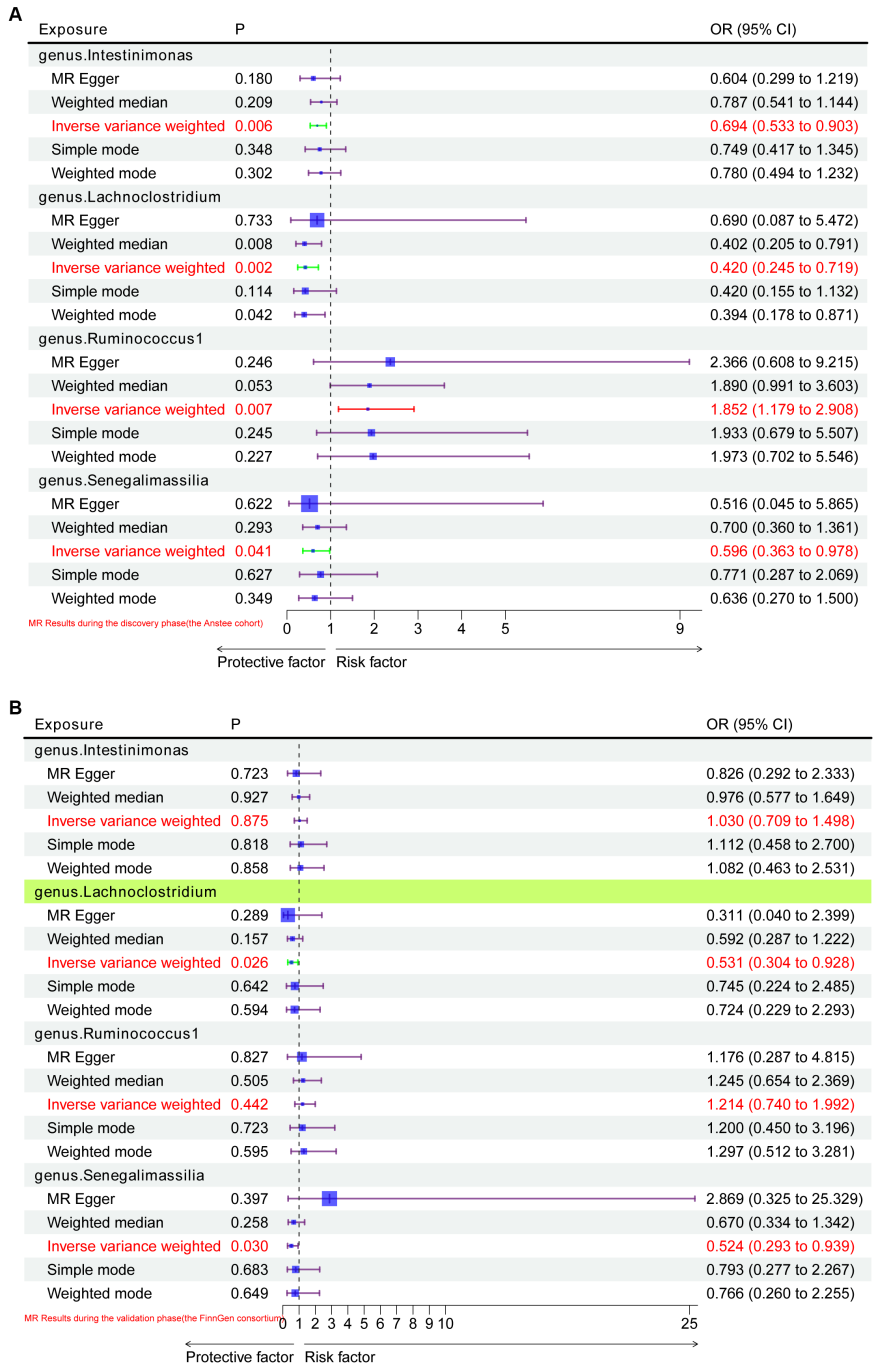
MR results of causal relationships between the gut microbiota and NAFLD in the discovery and validation datasets. **(A)** MR results of causal relationships between the gut microbiota and NAFLD in the Anstee cohort; **(B)** validation of positive results in the Anstee cohort by using FinnGen Consortium data.

In the discovery stage by using the Anstee cohort, gene prediction results showed that at the genus level, four gut microbiota were causally associated with NAFLD. A higher genetically predicted *Intestinimonas* (OR: 0.694, 95%CI: 0.533–0.903, *p* = 0.006, IVW), *Lachnoclostridium* (OR: 0.420, 95%CI: 0.245–0.719, *p* = 0.002, IVW), and *Senegalimassilia* (OR: 0.596, 95%CI: 0.363–0.978, *p* = 0.041, IVW) were associated with a lower risk of NAFLD (Figure 2A). Contrastingly, *Ruminococcus1* (OR: 1.852, 95%CI: 1.179–2.908, *p* = 0.007, IVW) was associated with a higher risk (Figure 2A).

**Figure 3:**
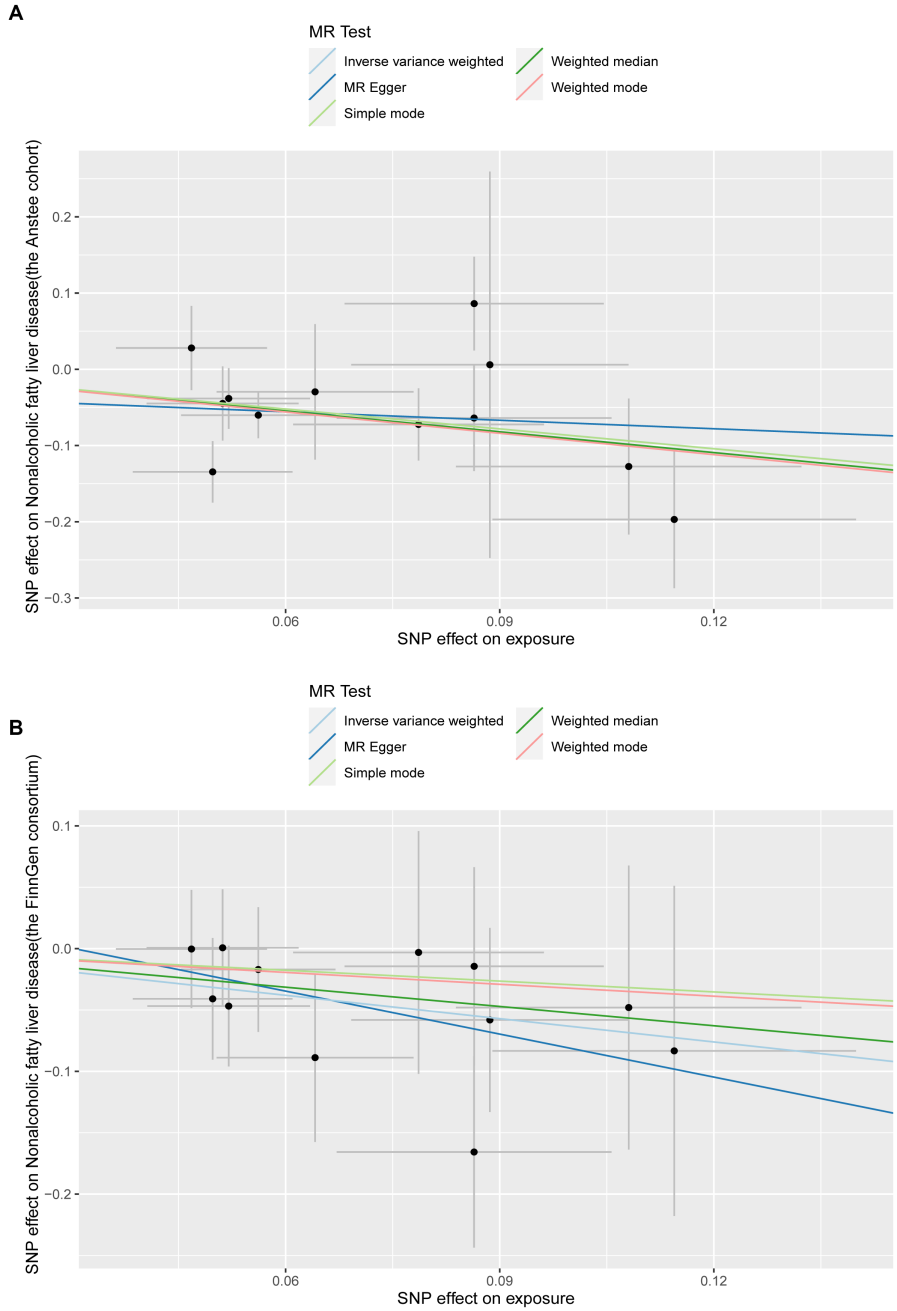
Scatter plots of the *Lachnoclostridium* genus positively associated with NAFLD in the discovery and validation datasets. **(A)** Anstee cohort; **(B)** FinnGen Consortium.

In the replication stage, by using the FinnGen consortium, we identified *Lachnoclostridium* (OR: 0.53, 95%CI: 0.304–0.928, *p* = 0.026, IVW) to be causally related to the risk of NAFLD with similar direction from the above four risk factors (Figures 2B, 3). Additionally, because the direction of the MR-Egger method was inconsistent with that of the IVW method (Figure 2B), we deemed that the relationship between the *Senegalimassilia* genus and NAFLD requires further investigation.

### 3.2 Sensitivity analysis

**Figure 4:**
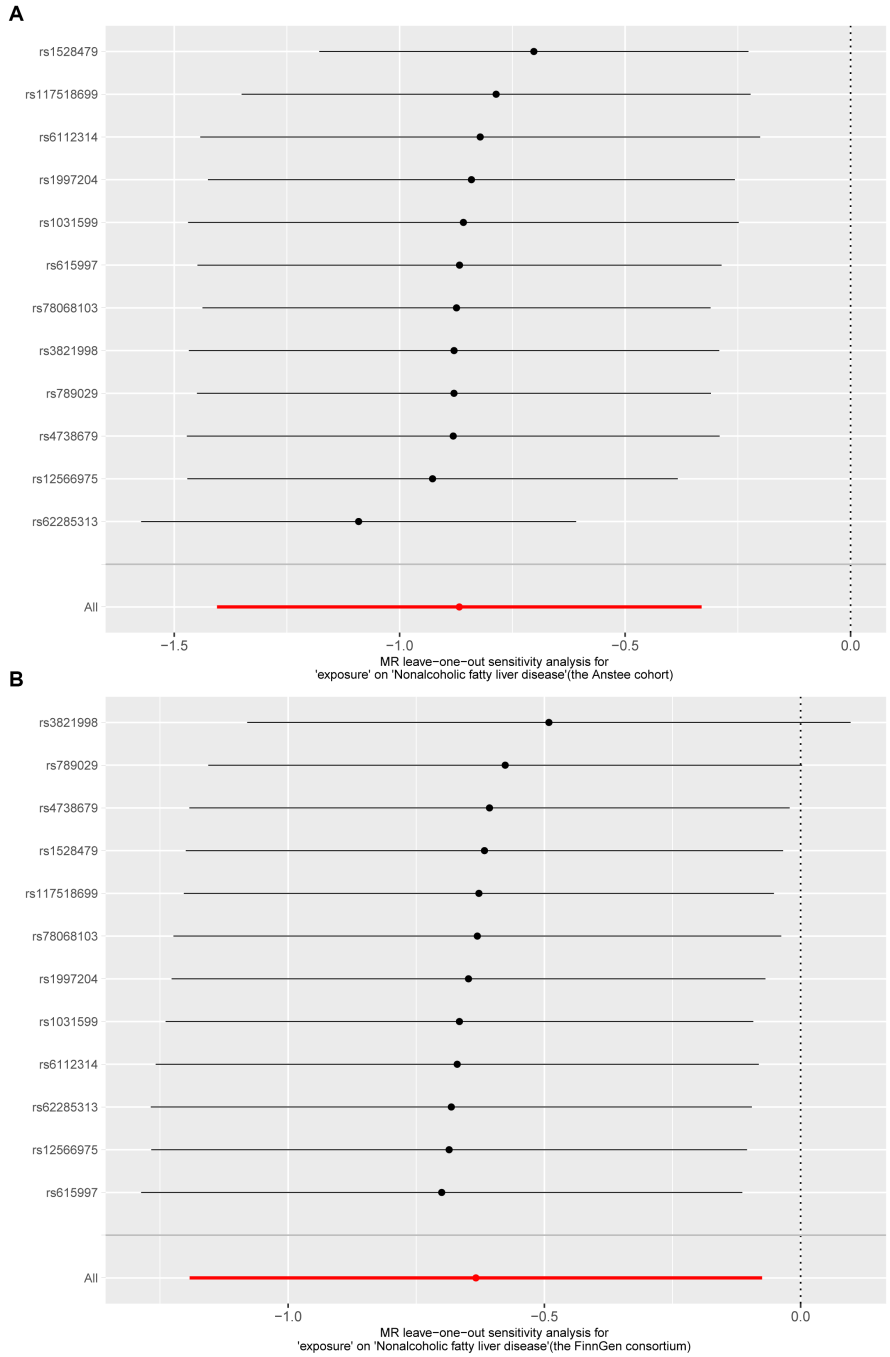
Results of “Leave-one-out” sensitivity analysis in the discovery and validation datasets. **(A)** Anstee cohort; **(B)** FinnGen Consortium.

We then tested the heterogeneity and pleiotropy of the *Lachnoclostridium* genus in the Anstee cohort and FinnGen consortium simultaneously. Cochran’s *Q* test showed that the MR analyses of the *Lachnoclostridium* genus had no heterogeneity in these two datasets (Table 1). The MR-Egger intercept test also showed that there is no pleiotropy in these two datasets (Table 1). Finally, the leave-one-out method demonstrated that the *Lachnoclostridium* genus achieved stable results after excluding each SNP individually, indicating that no single SNP had an exorbitant influence on the overall estimations (Figure 4).

**Table 1:** The heterogeneity and pleiotropy of the genus *Lachnoclostridium* in Anstee cohort and FinnGen consortium.

Datasets	Gut microbiota (exposure)	Heterogeneity		Horizontal pleiotropy		
		Cochran’s *Q*	*p* value	Egger intercept	SE	*p* value
Anstee cohort	*Lachnoclostridium*	15.27945	0.1700578	−0.03344837	0.06853461	0.6360377
FinnGen consortium	*Lachnoclostridium*	4.135252	0.9657921	0.03545706	0.06656826	0.6059139

### 3.3 Meta-analyses based on Anstee and FinnGen

**Figure 5:**
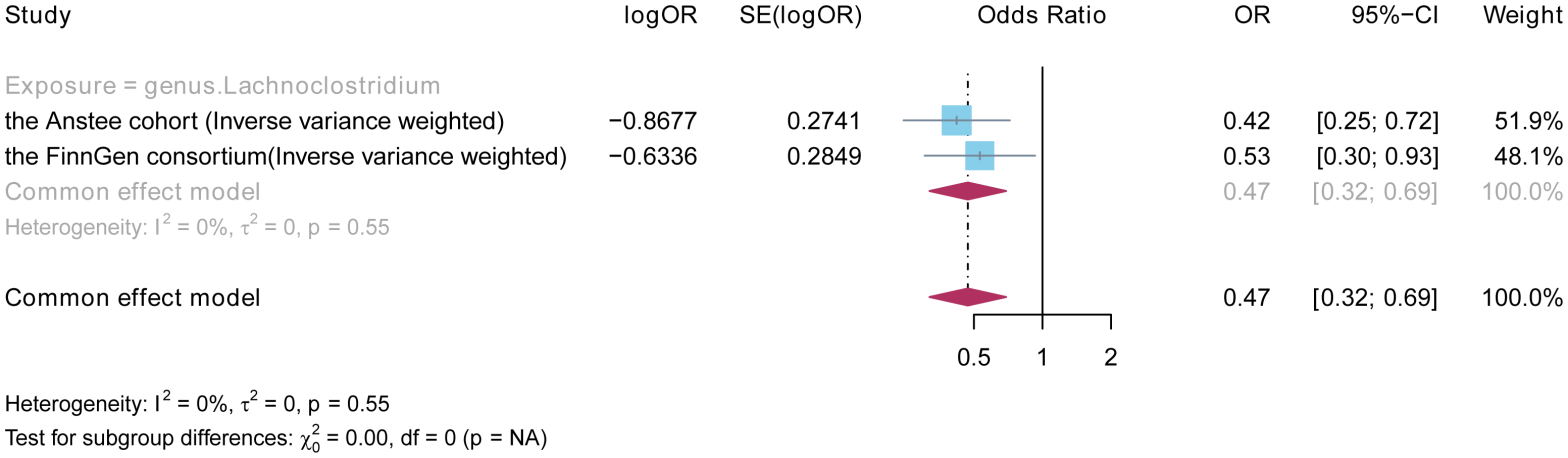
Fixed effects meta-analysis to verify the robustness of the causal relationship between the *Lachnoclostridium* genus and NAFLD.

To further demonstrate the robustness of the causal relationship between the *Lachnoclostridium* genus and NAFLD, we combined GWAS datasets from the Anstee cohort and the FinnGen consortium (2,377 European cases and 235,679 European controls) to perform a meta-analysis of the IVW results. The total effect size and confidence intervals were calculated by using a fixed-effects model. No heterogeneity was observed between the two cohorts. Moreover, the result showed that the *Lachnoclostridium* genus (OR: 0.470, 95%CI: 0.319–0.692, *p* = 0.0001, IVW) remained significant in the meta-analysis (Figure 5). By combining the results of two independent studies, a meta-analysis can increase statistical efficacy, strengthen the level of evidence, and improve the accuracy and reliability of this study.

## 4 Discussion

A series of observational studies have indicated that an imbalance in gut microbiota may contribute to NAFLD. Nonetheless, the real causal relationship between the human gut microbiota and NAFLD remains challenging to ascertain, owing to the inherent defects in observational studies and human ethical issues in experimental studies. TSMR analysis is based on the Mendel law of independent inheritance of gene variations, which can evaluate the potential causal relationship between exposure and outcome while avoiding the time-consuming and costly issues associated with RCTs. Thus, we explored the relationship between gut microbiota and NAFLD risk by the TSMR method, which is a natural RCT, using publicly shared large-scale GWAS data. Our results provide new evidence of the causal relationship between the *Lachnoclostridium* genus and NAFLD.

*Lachnoclostridium*, a genus of Firmicutes in the family Lachnospiraceae, is known to produce butyrate with anti-inflammatory properties and enhance the intestinal barrier by upregulating the tight junction protein (Vital et al., 2014; Mills et al., 2019). Endo et al. proved that butyrate-producing probiotics reduce non-alcoholic fatty liver disease progression in rats (Endo et al., 2013). In addition, previous animal experiments have shown that high-fat-fed mice induce hepatic steatosis with a significant increase in the relative abundance of *Lachnoclostridium* (Rondina et al., 2013; Li et al., 2018; Duan et al., 2019; Zhou et al., 2023). In our study, a lower genetically predicted *Lachnoclostridium* was associated with a higher risk of NAFLD. Thus, we speculated that the decreased fraction of *Lachnoclostridium* in the gastrointestinal tract might participate in the pathogenesis of NAFLD. However, the role of *Lachnoclostridium* in the pathophysiology of NAFLD requires further investigation.

This study has several strengths. The use of MR reduced the interference of confounding factors and false causality in the results. Our results offer a theoretical foundation for subsequent investigation of the regulatory mechanism of *Lachnoclostridium* in NAFLD. Resolving the mechanisms of *Lachnoclostridium* in NAFLD could help to identify methods to increase the abundance of *Lachnoclostridium* in the gut microbiota, optimize existing treatment approaches, or avoid potential side effects. Second, the current analysis made full use of the two independent population-scale GWAS data for NAFLD, making our study reliable and robust. Third, this discovery promotes potential interventions or therapies, such as new oral administration of probiotics or FMT, for the treatment of NAFLD. Increasing the relative abundance of *Lachnoclostridium* may effectively regulate the imbalance of gut microbiota, reduce gut permeability, and alleviate inflammatory responses, thereby preventing the progress and deterioration of NAFLD.

Our study has some limitations. First, the dataset we used included only a European population. Although using a single European population to investigate causal relationships can minimize population stratification bias, the results may not be generalizable to other populations. To address this limitation, GWAS data from patients with NAFLD of other races should be included in cross-racial MR analyses in future. Second, the original study on gut microbiota lacked GWAS summary statistics at the species level. Third, although we have confirmed a causal relationship between *Lachnoclostridium* and NAFLD, the mechanism of how *Lachnoclostridium* works remains unclear and requires further study. Fourth, owing to the use of different study populations, research designs, sample sizes, and measurement criteria in various studies, there may be inconsistencies in the data. Therefore, the results of the meta-analysis should be interpreted with caution. Fifth, there are limitations (data quality, sample size, genetic heterogeneity, and environmental factors) and biases (such as selection bias, information bias, and publication bias) in the use of publicly available GWAS data, which require cautious use.

## 5 Conclusion

Our MR study confirmed a potential causal relationship between the *Lachnoclostridium* genus and NAFLD, suggesting that augmenting the relative abundance of the *Lachnoclostridium* genus may be beneficial for NAFLD. This finding has promoted innovative interventions and new oral administration of probiotics or FMT as a means to restore healthy gut microbiota, thereby reducing the risk of NAFLD. However, further development of new probiotics and evaluation of their clinical efficacy are urgently required.

## Data availability statement

The original contributions presented in the study are included in the article/Supplementary Material, further inquiries can be directed to the corresponding authors.

## Author contributions

WD: Data curation, Formal analysis, Writing – review & editing. DC: Formal analysis, Writing – original draft. SZ: Formal analysis, Writing – original draft. AL: Conceptualization, Formal analysis, Supervision, Writing – review & editing. JX: Data curation, Formal analysis, Writing – review & editing. JZ: Conceptualization, Formal analysis, Supervision, Writing – review & editing.

## Footnotes

^1^ https://gwas.mrcieu.ac.uk/
